# Editorial: Cardiometabolic disease and psychiatric disorders

**DOI:** 10.3389/fpsyt.2023.1174055

**Published:** 2023-03-31

**Authors:** Giacomo Deste, Carlo Mario Lombardi

**Affiliations:** ^1^Department of Clinical and Experimental Sciences, University of Brescia, Brescia, Italy; ^2^Department of Medical and Surgical Specialties, Radiological Sciences and Public Health, University of Brescia, Brescia, Italy

**Keywords:** psychiatry, cardiology, cognition, COVID, heart failure, depression, Takotsubo, infarction

An increasing number of scientific evidences is showing comorbidity between mental disorders and cardiometabolic conditions as an insidious challenge for today medicine. The studies included in this Research Topic have addressed different aspects of this complex interaction, underlining the peculiar fragility of a “new” kind of patient. The increased workload during COVID-19 pandemic became the fuse for developing myocardial infarction with signs of heart failure, in a woman suffering from anxiety and arterial vasculitis (Pires et al.). Different inflammatory patterns explain the coexistence of arteritis, myocardial perfusion alterations due to valve regurgitation and the cascade of hormonal and neurotransmitter alterations related to anxiety. In fact, acute stressors might influence the structural stability of cardiac muscle and vessels, and increase their vulnerability to pathogenic factors. That's the case of Takotsubo syndrome: coronary vasospasm could be influenced by both inflammatory and psychiatric conditions, through an increased production of catecholamines and greater sensitivity of the myocardium to their effect. If there are clear evidences about the association between having a cardiometabolic disease (CMD) and the increased risk of developing depression, what about having simultaneous, different CMDs? A study included in the issue (Gong et al.) showed that cardiometabolic multimorbidity increases the likelihood of developing depression with more severe symptoms. The concomitant presence of different CMDs is thus associated with worse prognosis, requiring special attention to mental health status. Another study (Zhao et al.) further explored the role of depressive symptoms in post-stroke patients. Stroke is one of the most debilitating diseases of our time: it has deleterious effects on quality of life, due to frustrating disabilities, cognitive impairment, and reduced autonomy. Depressive symptoms are often found in post-stroke patients and are correlated with basic functions disability and perceived physical pain. A relevant proportion of stroke patients showed symptoms of depression, with a higher prevalence in females living in rural areas, while in males a stronger association between functional limitations on daily activities and depression emerged. Post-stroke depression is emerging as a new, common condition, pointing to the importance of properly assessing pain and depressive symptoms in post-stroke patients. Another condition that is often found among patients with psychiatric disorders is Metabolic Syndrome (MS), which is well-known to be highly prevalent among psychiatric patients, in particular in those living with schizophrenia or mood disorders. Insulin Resistance (IR) is closely involved in MS's development and both these conditions have a role in predicting cardiovascular risk. An Italian research team (di Girolamo et al.) found that a small but significant proportion of patients diagnosed with ADHD suffers from MS. ADHD increases the risk of obesity and is also associated with type 2 diabetes. Many people with ADHD have unhealthy dietary habits, including late-night eating and irregular intervals between meals. The authors recommended to screen ADHD patients for MS components, paying particular attention to blood triglycerides concentration, diastolic blood pressure and waist circumference, as they may predict the risk of a full-blown metabolic syndrome. Another critical element of interest about patients suffering from CMDs is the impact of their health condition on cognitive performance. According to another study (Niu et al.), patients with Heart failure (HF) tend to show poorer performance on cognitive tests, compared to individuals without heart failure. HF is indeed highly correlated with Cognitive Impairment (CI), and to an increased risk of dementia and Alzheimer's disease. Patients with HF may show poorer short-term memory and difficulties with concentration, resulting in reduced medication compliance and other self-care activities. This can lead to higher rates of readmission and increased mortality. In this context, another study (Raei et al.) suggested that an appropriate and early cardiac rehabilitation may play an essential role in managing anxiety and stress symptoms in post-myocardial infarction patients. Myocardial infarction (MI) has serious social, medical and psychological consequences, that may pose a relevant threat to patients' mental health. Nonetheless, a bilateral causality between mental and cardiometabolic diseases has been recognized: if the occurrence of psychic symptoms in patients with cardiometabolic diseases is common, the increased risk of heart disease in psychiatric patients is now demonstrated, with solid evidences. A large proportion (up to 66%) of patients with acute myocardial infarction develops anxiety symptoms, increasing their overall chances of mortality. Additional symptoms such as asthenia, loss of energy, sleep disturbances, and chest pain can further restrict their daily activities, leading to a vicious circle of adverse consequences, in the progression of the disease. Cardiac rehabilitation (CR) serves as a vital tool in addressing CMDs in their wholeness. The authors explored how CR conducted with the FCEM (Family-Centered Empowerment Model) approach can lead to psychological amelioration in patients with MI. Family members could have a direct impact in mitigating patients' psychological problems, making the FCEM-CR a valid tool in improving quality of life. All these interesting findings shed new light on an important topic that is becoming everyday more relevant for the clinical practice and for the life of people living with cardiometabolic conditions and psychiatric disorders.

## Author contributions

All authors listed have made a substantial, direct, and intellectual contribution to the work and approved it for publication.

